# Insight into pathway of monosaccharide production from integrated enzymatic hydrolysis of rice straw waste as feed stock for anaerobic digestion

**DOI:** 10.1038/s41598-023-27398-6

**Published:** 2023-01-04

**Authors:** Piyathida Khantibongse, Chavalit Ratanatamskul

**Affiliations:** 1 Interdisciplinary Program of Environmental Science, Graduate School, Chulalongkorn University, Bangkok, Thailand; 2 Department of Environmental Engineering, Chulalongkorn University, Bangkok, Thailand; 3 Research Unit On Innovative Waste Treatment and Water Reuse, Faculty of Engineering, Chulalongkorn University, Phayathai Road, Pathumwan, Bangkok, 10330 Thailand

**Keywords:** Plant sciences, Environmental sciences

## Abstract

This research examined the possible pathway of monosaccharide production from the rice straw waste using three integrated enzymatic hydrolysis approaches: boiled hot water pre-treatment with enzyme, alkaline pre-treatment with enzyme, and acid pre-treatment with enzyme, that can be further used as the feedstock for anaerobic digestion. Two cellulase enzymes: SIGMA-ALDRICH laboratory grade cellulase from *Aspergillus niger* and atres Zymix plus as a commercial cellulase enzyme were applied. It was found that the boiled hot water pre-treatment with the commercial cellulase gave the highest total monosaccharides yields. Glucose was the most significant part (78–86%) of the monosaccharides. For the pre-treatment with dilute acid, glucose was also the main component of monosaccharides; however, for the alkali pre-treatment, xylose was the main monosaccharide. It made up 48–85% of the total monosaccharide compared to glucose that made up 5–49% of total monosaccharide. Boiled rice straw with commercial cellulase enzyme provided the highest glucose yield compared to other experiments. Moreover, the obtained results from GC–MS/MS analysis show that up to 62 species of phenolic compound could be found in enzymatic hydrolysis of the rice straw waste. Aromatic and aliphatic hydrocarbon substances were also detected in the FEEM analysis. From the overall results, the integrated enzymatic hydrolysis with boil hot water pre-treatment was the most efficient method for monosaccharide production from the rice straw waste.

## Introduction

Rice is an important food crop that is cultivated in large amount in many parts of the world. Global production of rice straw was reported to be 685.24 million ton^1^. In 2018, the rice harvesting area in Thailand was 8.9 million ha with the production of 25.18 million tons of paddy. Inappropriate rice straw waste management has caused significant environmental problems after the harvesting. Approximately 50% of rice straw was burnt in the field, causing PM 2.5, which is a major source of air pollution and global warming problems^2^.

Rice straw is a lignocellulosic material which can be converted to energy by anaerobic digestion. Most studies on the valorization of agricultural residues in Thailand have been focused on composting and ethanol production. However, enhanced digestibility of rice straw is needed for anaerobic digestion process since the rice straw contains cellulose, hemicellulose, and lignin, which are slowly biodegradable organic matters. Hydrolysis of these materials might be concerned as the first step for either digestion to biogas. The pre-treatment of lignocellulosic waste must be considered before anaerobic digestion process. Lignocellulosic biomass can be pretreated by many pre-treatment techniques including physical, chemical, and biological techniques such as milling, steam explosion, ammonia fiber explosion (AFEX), diluted and concentrated-alkaline/acid hydrolysis, and biological pre-treatment^3^. The cellulose in a plant consists of the parts with crystalline structures, and the parts with not well-organized, amorphous structures^4^. The cellulose strains are ‘bundled’ together and formed so called cellulose fibrils or cellulose bundles. These cellulose fibrils are mostly independent and weakly bound through hydrogen bonding^4^. The dominant component of hemicellulose from agricultural plants, like grasses and straw, is xylan^5^. Furfurals and hydroxymethyl furfurals are by-products of hemicellulose degradation^6^**.**

Lignin which provides plant structural supports is impermeable and non-water soluble. It resistances against microbial attack and oxidative stress. It was a major deterrent to enzymatic and microbial hydrolysis of lignocellulosic biomass^7^. Mechanical pre-treatment can increase porosity and reduces crystallinity of cellulose and degree of polymerization. However, size reduction process consumes high energy^8^. Hydrothermal pre-treatment is a relatively mild method that does not require any catalysts and does not cause significant corrosion problem. Dilute acid pre-treatment has been extensively investigated and developed to pretreat lignocellulosic biomass for fuel production^9^. Acid pre-treatment with HCl, H_2_SO_4_ results in improvement of enzymatic hydrolysis since it causes the solubilization of hemicellulose and the reduction of cellulose^10^. Alkali pre-treatment also can result in hydrolysate with higher COD concentration and better cellulose degradation, but gas production was lower than that of acid hydrolysate. During the hydrolysis of lignocellulosic biomass, various byproducts also can be formed such as furan derivatives and phenolics. Compared to acid and hydrothermal processes, mild alkaline pre-treatment leads to less solubilization of hemicelluloses and less formation of inhibitory compounds, and they can be operated at lower temperatures. NaOH and KOH are the most commonly used forms of alkali, but their cost is a serious limitation^11^.

The releasing of monosaccharide from enzymatic hydrolysis of rice straw waste might be challenging as the feedstock for anaerobic digestion. Enzymatic digestibility of biomass depends on the enzyme concentration to convert the carbohydrate polymers into monomeric sugars^12^. Badiei et al.^13^ suggested that selection of pre-treatment method should consider sugar yield, avoidance of degradation of sugars and minimizing the formation of inhibitors for further fermentation process. Alkali pre-treatment can result in high sugar yields, but operation cost is high. The complete release of sugar increased the pore volume of the pretreated solid residues, resulted in an efficiency of 70% for the enzymatic hydrolysis^14^. The dilute acid pre-treatment was reported to effectively hydrolyze hemicelluloses to xylose for the fermentation process^5^. Talebnia et al.^15^ suggested that addition of xylanase enzyme in the wheat straw digestion could improve the final yields of sugars. The glucose production during enzyme hydrolysis had variations largely due to the different lignin contents and enzyme accessibility to waste structure^16,17^. Therefore, more research is needed to enhance the enzyme hydrolysis purpose.

For this current work, we aimed to investigate the platform of monosaccharide production from the rice straw waste as the feedstock for anaerobic digestion. Few research works focused on the platform of monosaccharide that is released from the rice straw waste using the integrated enzymatic hydrolysis method. Moreover, the insight into pathway of monosaccharide production from the rice straw waste by the integrated enzymatic hydrolysis method is still not completely described. Three integrated enzymatic hydrolysis approaches: hot water pre-treatment with enzyme, alkaline pre-treatment with enzyme, and acid pre-treatment with enzyme, were considered. It is also necessary to explore the suitable pre-treatment method with short treatment period (4 h) to enhance digestibility and its effectiveness on enzymatic hydrolysis of rice straw waste. Moreover, the possible pathway of monosaccharide production from the rice straw waste degradation is proposed in a simplified flow diagram to better understand the possible mechanism from the obtained results using various instrumental analysis tools such as HPLC and FEEM measurement techniques for further practical application.

## Experimental methods

### Enzyme and chemicals

This research investigated the integrated enzymatic hydrolysis of rice straw waste using two cellulase enzymes: a laboratory-grade enzyme from *Aspergillus niger* (SIGMA-ALDRICH from Merck KGaA, Germany) and a commercial enzyme (atres Zymix plus from Atres group, Germany). The commercial enzyme is a mixture of cellulase, xylanase, endo-1,4, Alcohols, C16-18,ethyloxalated, and 1,2-benzisothizaol-3(2H)-one. In this research, diluted (0.1 N) sulfuric acid (H_2_SO_4_) and sodium hydroxide (NaOH) were used for acid and alkali pre-treatments at room temperature. Enzymatic hydrolysis time of 48 h was applied in all experimental investigation. Suitable pre-treatment method with enzymatic hydrolysis under practical operating condition for large scale system was identified based on sugars production in the final substrate.

### Collection and preparation of rice straw waste

Rice straw wastes were collected from Hom–Mali rice paddy fields in Lop Buri and Nakhon Pathom provinces of Thailand. Permissions were obtained from the farm owners for collecting and using the rice straw waste in this study. Our study also complies with relevant institutional, national, and international guidelines and legislation. The samples were mixed and then dried in the oven at 105 °C for 24 h and were chopped and grinded to an average size of 1–2 cm. The samples were mixed and kept in sealed plastic bags at room temperature for experimental investigation. Table 1 illustrates the composition of Hom–Mali rice straw.

**Table 1:** Composition of Hom–Mali rice straw used in this study.

Composition	Dry matter (%)
Lignin	15.61
Alpha-cellulose	36.7
Hemicellulose	20.21
Extractives	18.74
Other	8.74

### Pre-treatment methods and experimental procedures

Rice straw wastes were pretreated prior to anaerobic digestion with one sole enzymatic hydrolysis method (with DI water) and three integrated enzymatic hydrolysis methods: (1) alkaline pre-treatment followed by enzymatic hydrolysis; (2) acid pre-treatment followed by enzymatic hydrolysis; and (3) boiled hot water pretreatment followed by enzymatic hydrolysis. Dried rice straw waste samples of 5 g were used for each experimental run. Experimental investigation by enzymatic hydrolysis and integrated enzymatic hydrolysis methods is shown below (Table 2).

**Table 2:** Experimental investigation for four different pre-treatment methods of integrated enzymatic hydrolysis.

	Chemical pre-treatment			Enzymatic hydrolysis			
	Chemical	Temp	Time	LR grade Cellulase dosage	Commercial grade cellulase dosage	Temp	Time
1	200 ml DI water	Room temp,	4 h	2.5, 5.0, 7.5 Units	500, 1000, 1500, and 2000 µL	55ºC	48 h
2	200 ml of 0.1 N H_2_SO_4_						
3	200 ml of 0.1 N NaOH						
4	200 ml DI water	95–100 ºC					

After the diluted acid and diluted alkaline pre-treatments, samples pH was adjusted to 5–6 using concentrate NaOH and H_2_SO_4_, respectively.

Glucose plays important roles in anaerobic digestion for biogas production. Glucose yield can be calculated by Eq. (1).

1 $$\mathrm{Glucose\; yield }\left(\mathrm{\%}\right)= \frac{\mathrm{glucose\; weight\; in\; digestate }\times 100}{\mathrm{Substrate\; } (\mathrm{rice\;straw})\; \mathrm{ dry\; weight}}$$

### Analytical methods

The surface morphology of rice straw was investigated using scanning electron microscope (SEM). Spectrofluorometer (Jasco FP 6200) Fluorescence excitation-emission matrix (FEEM) was used to study the compositions of soluble organic matters in the liquid digestate in each treatment. Excitation wavelength range were 220–600 nm and emission wavelength range were 250–700 nm**.** Pentosan in solid residues after the pre-treatments and enzymatic hydrolysis were also analyzed by TAPPI standards (TAPPI T223). The liquid hydrolysate from all samples were analyzed for monosaccharides production using HPLC (Alliance e2695b, Shodex Asahipak NH2P-50 4E column 4.6 mm ID × 250 mm L).

## Results and discussion

### Effect of chemical pre-treatment on the change in surface morphology of rice straw waste

**Figure 1:**
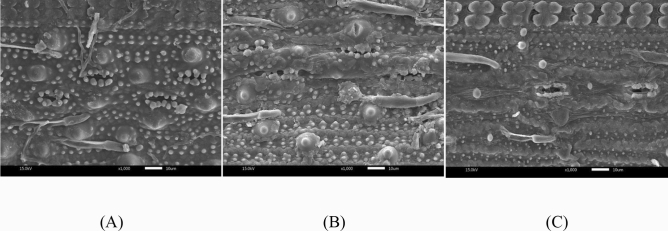
Rice straw surface morphology at × 1000 (**A**) Untreated rice straw, (**B**) Rice straw treated with 0.1 N H_2_SO_4_ for 4 h, (**C**) Rice straw treated with 0.1 N NaOH for 4 h.

The comparative morphology was conducted to observe the outward appearance of untreated and pretreated rice straw waste. Surface morphology of rice straw waste was investigated by scanning electron microscope (SEM). The morphology of rice straw waste before and after chemical pre-treatments are illustrated (Fig. 1). Significant morphological changes of tissue and texture were observed. Figure 1B illustrates that the surface morphology of the acid pretreated rice straw after 4 h had smoother surface and less silica on the surface and more damaged surface compared to the untreated one, which is shown in Fig. 1A. The presence of silica at the outer layer of rice straw can reduce enzymatic hydrolysis activity^18^. In Fig. 1C, rice straw which was pretreated with 0.1 N NaOH for 4 h shows the dramatically change in the surface appearance and silica removal compared to the untreated rice straw in Fig. 1A. Dumbbell-shaped silica is also clearly observed in Fig. 1C as well.

### Analysis of volatile organic matters in the liquid digestate by GC–MS/MS

Liquid digestate from the integrated enzymatic hydrolysis methods were analyzed using GC–MS/MS to observe volatile organic matters. Different pre-treatment methods resulted in different volatile organic compounds such as aromatic hydrocarbon and phenolics compounds. The processes with highest dosage of enzyme (2000µL commercial enzyme) were chosen for the analysis.

**Figure 2:**
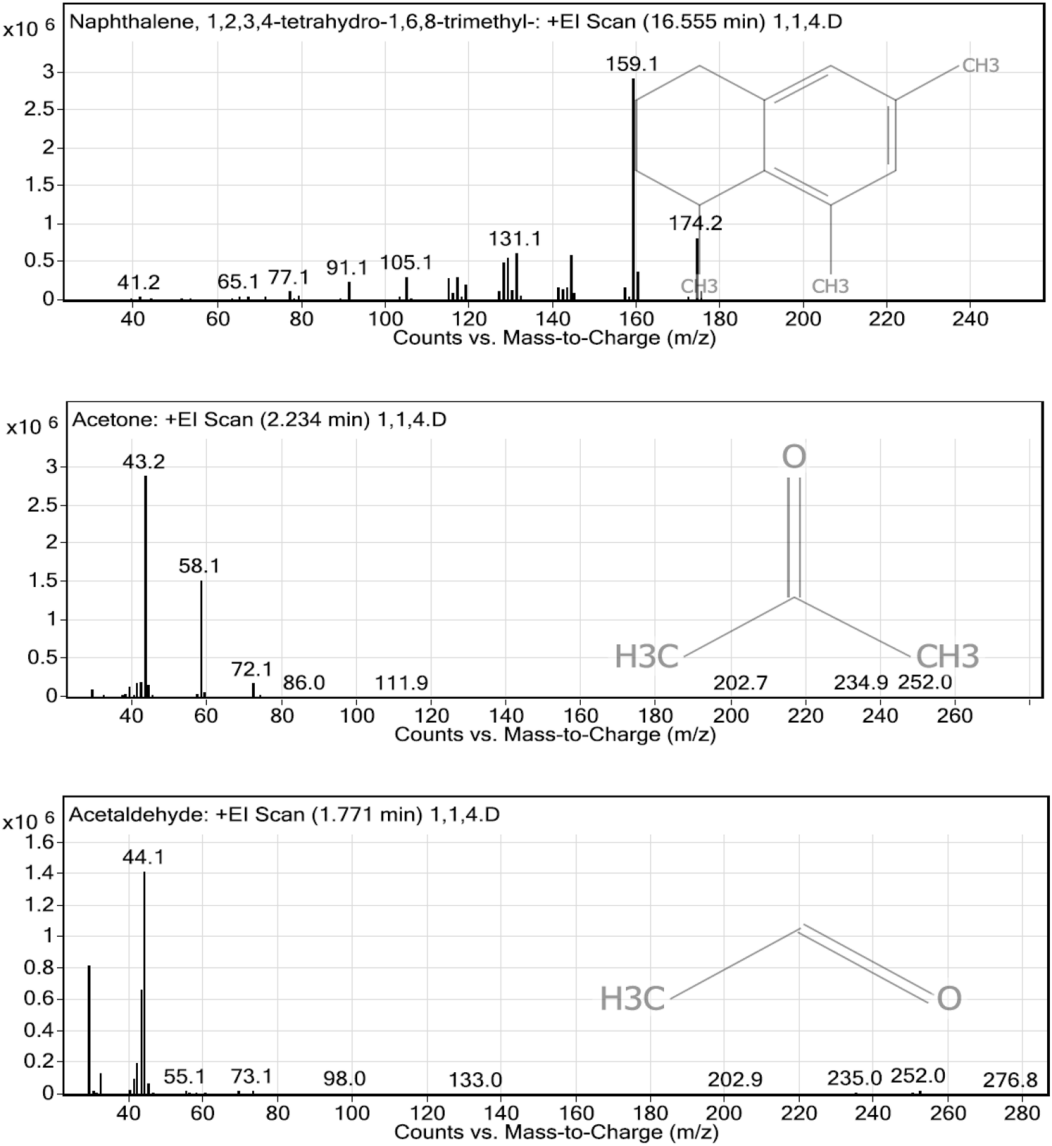
The most abundant volatile organic matters from the enzymatic hydrolysis of untreated rice straw waste.

The obtained results of the rice straw waste treated with the sole enzymatic hydrolysis shows a total number of 62 volatile organic matter species. Three most abundant volatile matter species were naphthalene, 1,2,3,4-tetrahydro-1,6,8-trimethylz, acetone, and acetaldehyde (Fig. 2). Polycyclic hydrocarbons such as naphthalene’s derivatives, benzenes and derivatives, acetic acids, furan and derivatives, furfural, phenols derivatives, and ester compounds were also detected.

**Figure 3:**
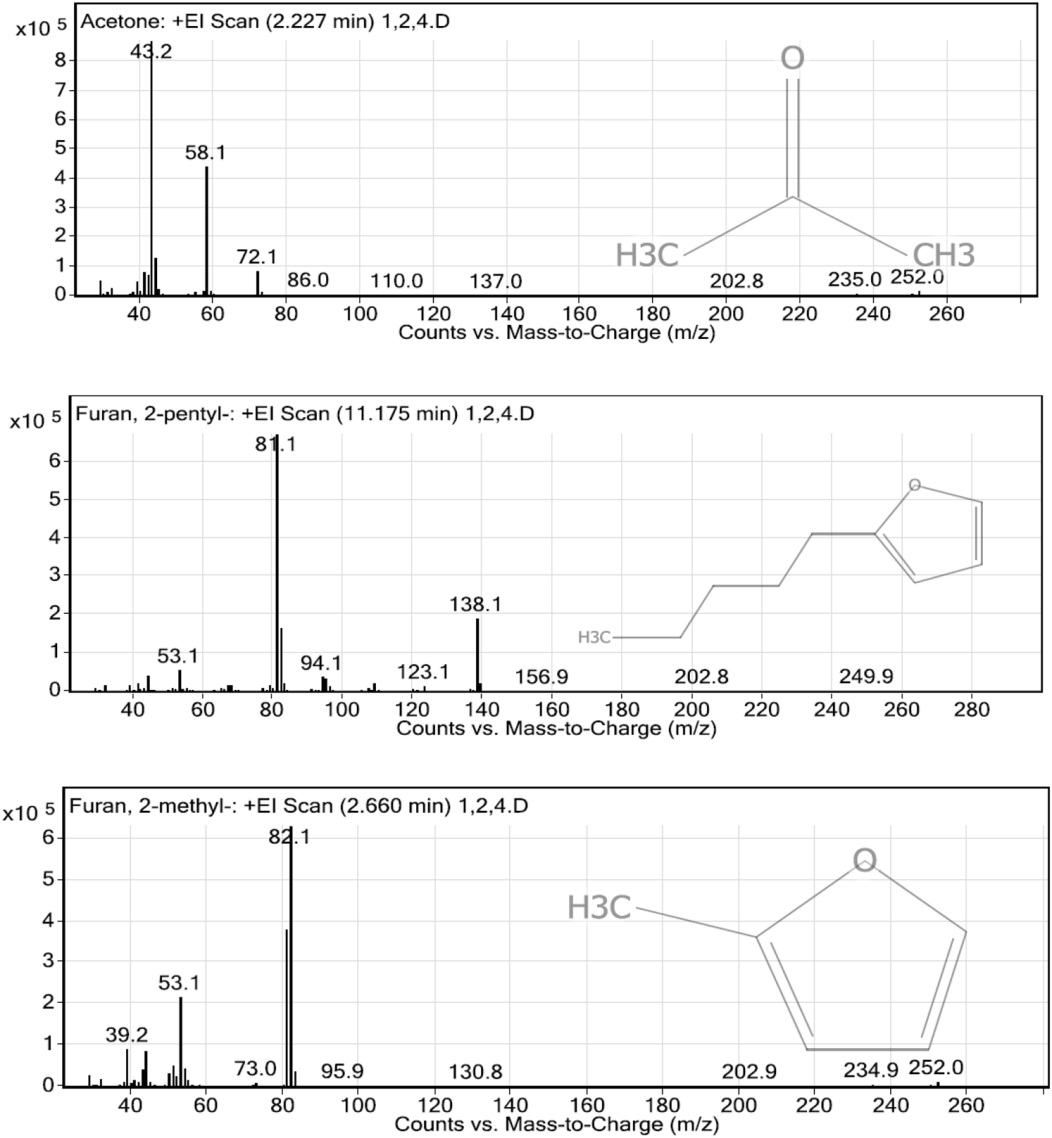
The most abundant volatile organic matters from acid pre-treated rice straw waste followed by enzymatic hydrolysis.

Twenty-two volatile organic matter species were found in the liquid fraction from acid pre-treated rice straw waste followed by enzymatic hydrolysis. The most abundant volatile organic matter species are acetone, furan,2-pentylz and furan,2-methylz (Fig. 3). Acetic acid, furfural, phenolic compounds, and ester compounds were also found.

**Figure 4:**
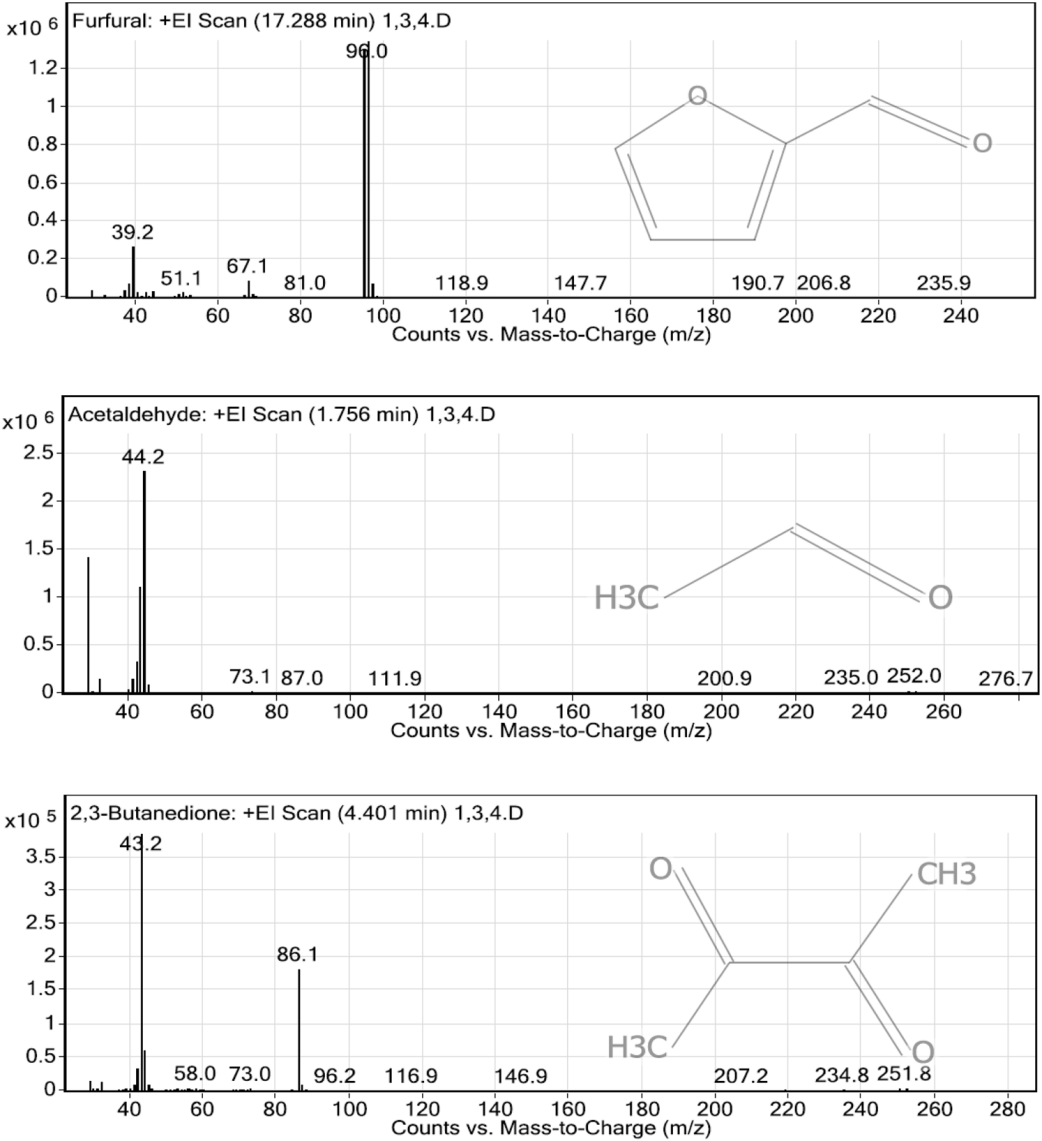
The most abundant volatile organic matters from alkaline pre-treated rice straw waste followed by enzymatic hydrolysis.

Liquid fraction from alkaline pre-treated rice straw waste followed by enzymatic hydrolysis had 17 species of volatile organic matter species. Acetaldehyde, furfural, and 2,3-butanedione were the most abundant volatile organic matter species (Fig. 4). Group of furans were mostly found from this pre-treatment method. Acetic acid, ethanol, and naphthalene were also found.

**Figure 5:**
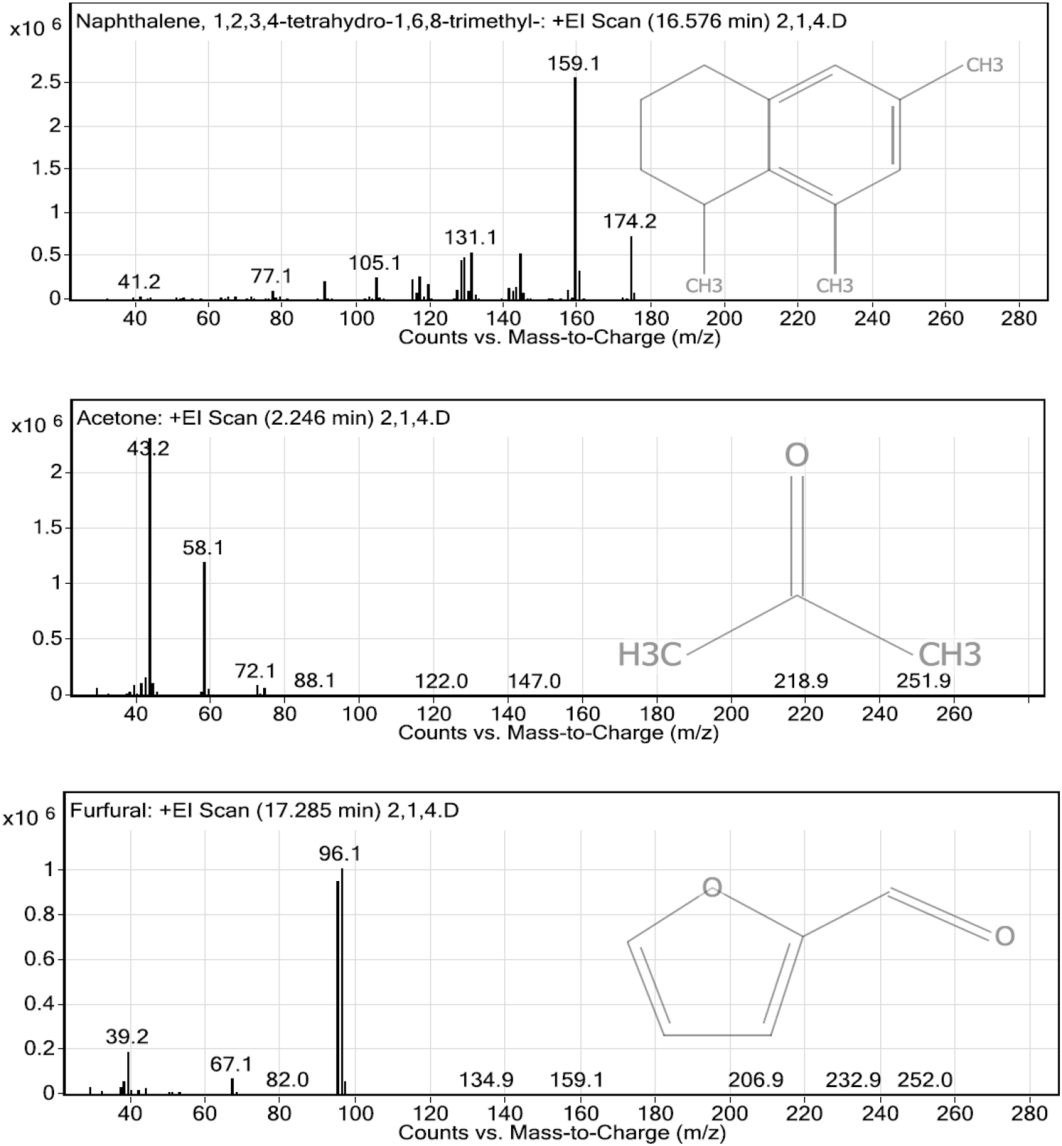
The most abundant volatile organic matters from boiled rice straw followed by enzymatic hydrolysis.

Forty-nine species of volatile organic matters were found in the liquid fraction from the boiled hot water pre-treatment of rice straw waste followed by enzymatic hydrolysis. The most abundant volatile matter species are Naphthalene, 1,2,3,4-tetrahydro-1,6,8-trimethylz, acetone, and acetaldehyde (Fig. 5). Groups of polycyclic hydrocarbons were mostly found such as cyclohexadiene and indenes. Acetic acid, phenolic compounds, furans, and furfural were also detected.

The obtained results from the GC–MS/MS analysis show that up to 62 species of phenolic compound could be found in enzymatic rice straw hydrolysis. In addition, 2,3-Butanedione substances, which can be reduced to 2,3-Butanediol, were also found in every pre-treatment in this study. Some studies mentioned that the reduced form of 2,3-Butanedione was a precursor in manufactures of butadiene, synthetic rubbers, plastics, and pesticides^19,20^. Phenolic phytochemicals are important aromatic secondary metabolites in plants, many of which are commonly substituted by sugar molecules such as glucose, arabinose, xylose, rhamnose and galactose^21^. Many of volatile products found in this study are useful. Acetic acid can be converted into biogas. However, benzene compounds, phenol derivatives, furans derivatives, and furfural derivatives may affect health, and some of them are microbial inhibitors. The rice straw waste pretreated with the sole enzymatic hydrolysis could generate the highest number of volatile organic matters (62 species), followed by the boiled hot water pre-treatment with enzymatic hydrolysis (49 species); acid pre-treatment with enzymatic hydrolysis (22 species); and alkaline pre-treatment with enzymatic hydrolysis (17 species). During the pre-treatment with acid and alkaline solutions, pHs were adjusted to provide suitable environment for enzyme activity.

### Dissolved organic compounds in liquid digestate

**Figure 6:**
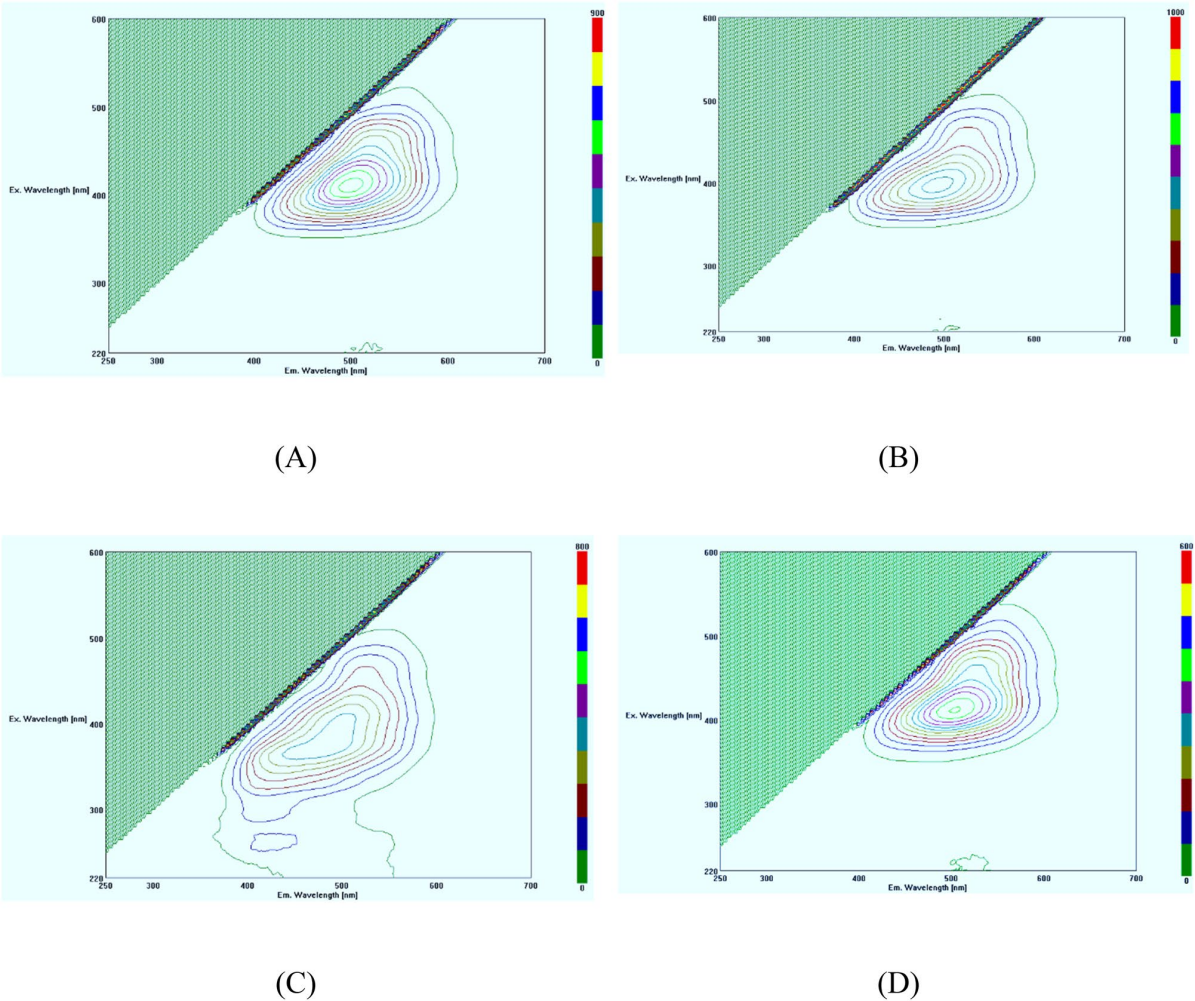
Fluorescence excitation-emission matrix (FEEM) spectra of humic acid-like and fulvic- like substances in liquid digestate from different pre-treatment with enzymatic hydrolysis. (**A**) is with sole enzymatic hydrolysis, (**B**) is acid pre-treatment followed by enzymatic hydrolysis, (**C**) is alkaline pre-treatment followed by enzymatic hydrolysis, (**D**) is boiled hot water pre-treatment followed by enzymatic hydrolysis.

FEEM analysis was used to investigate dissolved organic matters in the liquid digestate from the untreated and pretreated rice straw with enzymatic hydrolysis. Untreated and pretreated rice straw waste with 2000 µL commercial cellulase enzyme were chosen for the FEEM analysis. Peaks location were compared with Chen et al.^22^ as a reference. The results show that the fluorescent characteristics of liquid digestates from the untreated rice straw, dilute acid-pretreated rice straw, dilute alkaline-pretreated rice straw, and boiled rice straw followed by the commercial cellulase enzyme (Fig 6A–D respectively), had the same peak location. The location of peaks were identified as humic acid-like substances and small amount of fulvic acid-like substances (Region III). This implies that there was a generation of humic acid-like and small amount of fulvic acid-like substances from conversion of the rice straw into the digestate (Fig. 6).

Humic substances are weak acidic electrolytes with carboxylic- and phenolic-OH groups with a micelle-like structure and with a molecular weight between 500 (fulvic) and 100,000 (humic)^23^. Humic acids can coagulate when the strong-base extract is acidified while fulvic acids are soluble when the strong-base extract is acidified^24^. Humic acids are macromolecular and complex, and composed of substituted aromatic and aliphatic hydrocarbon core materials. The aliphatic hydrocarbons are less stable which may be better degradable. Humic acids has higher free radical compared to fulvic acids^25^. Humic and fulvic substances enhance plant growth directly through physiological and nutritional effects. Some of these substances function as natural plant hormones (auxins and gibberellins) and can improve seed germination, root initiation, uptake of plant nutrients and can serve as sources of N, P and S^26^. Humic acid-like and fulvic acid-like substances, which were detected by the spectrofluorometer analysis, were also the organic substances from the rice straw conversion using the enzymatic hydrolysis. The highest peak of humic acid-like substances was found in boiled rice straw with commercial cellulase enzyme (Fig. 6D) followed by the untreated rice straw with commercial cellulase enzyme (Fig. 6A), the acid pre-treated rice straw with commercial cellulase enzyme (Fig. 6B), and the alkaline pre-treated rice straw with commercial cellulase enzyme (Fig. 6C), respectively. The obtained results from FEEM analysis show the degradation of the rice straw waste into different groups of aromatic and aliphatic hydrocarbon substances that were also related to the GC–MS/MS results in section "Analysis of volatile organic matters in the liquid digestate by gc–ms/ms".

### Pentosan production

**Figure 7:**
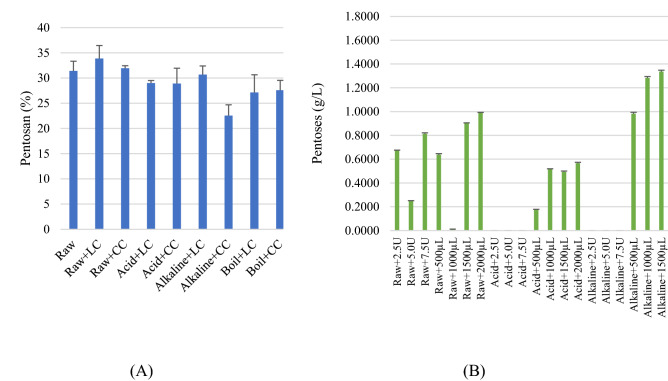
(**A**) Percentage of pentosan remaining in the pre-treated and hydrolysed rice straw. (**B**) Pentose production in the liquid digestate (LC is laboratory grade cellulase enzyme, CC is commercial grade cellulase enzyme).

Figure 7A shows the percentages of pentosan in the untreated and the pre-treated rice straw waste followed by enzymatic hydrolysis. The raw rice straw waste contains pentosan of 32%. The changes in pentosan content of rice straw waste was due to the attack by enzyme hydrolysis with the pre-treatment methods. Figure 7B shows the average pentose production in liquid digestate after the pre-treatment and enzymatic hydrolysis (LC: laboratory grade cellulase enzyme, CC: commercial grade cellulase enzyme). Samples with the highest dosage of both LC and CC were chosen. The obtained results suggested that the alkaline pre-treatment with commercial grade cellulase gave the lowest percentage of remaining pentosan in the digested rice straw, followed by boiled rice straw with enzymatic hydrolysis. In addition, the amount of pentose sugars released in liquid digestate by the same process (alkali pre-treatment) was greatest and that was associated with a decrease in the amount of pentosan in the rice straw waste. Higher pentoses production in the liquid digestate, in contrast, could result in lower remaining pentosan in samples. Hemicellulose in biomass can be digested to smaller units of monosaccharides such as xylose.

Pentosan consists of molecules of pentose, such as xylose, which was a precursor to furfural^27^ while cellulose is composed of only hexose (glucose) molecules. Pentose is a monosaccharide with five carbon atoms. They exist in open-chain (linear) and closed-chain (cyclic) forms which can be converted into each other form in water solutions^28^. The presence of less pentosan in the digested straw samples means that hemicellulose was more digested into monosaccharides due to hemicellulose contains both sugar molecules, pentoses and hexoses. The less remaining pentosan in the digested rice straw means hemicellulose in the sample was more digested to monosaccharides.

### Monosaccharides production

**Figure 8:**
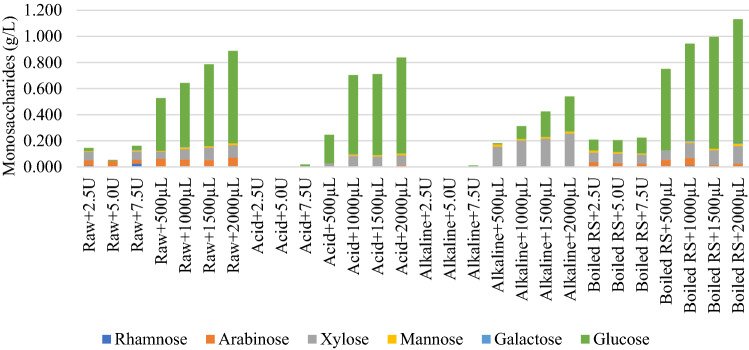
Detection of Monosaccharides production in liquid digestates.

Lignocellulosic substrates can be converted into biofuel. Firstly, organic matter in the rice straw waste is hydrolyzed to smaller molecules such as sugars, fatty acids, and amino acids by enzymes. After that, the products can be fermented by the acid-forming bacteria into short-chain alcohols, fatty acids, carbon dioxide, and hydrogen^29^. Figure 8 shows the characteristics of monosaccharides production in the liquid digestates which were obtained from different pre-treatment methods.

Figure 8 illustrates that total monosaccharide amounts increased as the quantity of cellulase enzyme increased for all cases of the pre-treatment methods. Boiled hot water pre-treatment with the commercial cellulase (atres Zymix plus) could achieve the highest amount of monosaccharides yields. An increase in the concentration of commercial cellulase from 500 to 2000 µL could significantly enhance the releasing of monosaccharide from the rice straw waste up to 1,100 mg/L (1.1 g/L). Glucose was the significant part (78–86%) of the monosaccharides for the pre-treatment with boiled hot water followed by commercial cellulose hydrolysis. Since cellulose and hemicellulose are the main compositions of lignocellulosic biomass. They comprise of mainly glucose. Therefore, glucose was the predominant composition of monosaccharides after the enzyme degradation. Glucose, mannose, xylose, arabinose and rhamnose were also found in the case of pre-treatment with boil hot water, followed by the enzymatic hydrolysis using the commercial enzyme.

Xylose was the second predominant type of monosaccharide that was detected in this case. However, the laboratory-grade cellulase enzyme with the concentration from 2.5 to 7.5 units seemed to be not sufficient to release the monosaccharide from the rice straw waste for all the integrated enzymatic hydrolysis methods (acid, alkaline, boiled water pretreatment methods with enzyme hydrolysis). The highest detected amount of monosaccharide was only 0.2 g/L with the case of boiled hot water pre-treatment and enzyme hydrolysis). Higher dosages of the laboratory-grade enzyme were required. However, the cost of laboratory-grade enzyme is higher than that of commercial grade ones. Therefore, the commercial grade enzyme is more promising in the real practice for enzymatic hydrolysis of rice straw waste. For the dilute acid pretreatment with enzyme hydrolysis, glucose was also the main component of produced monosaccharides, followed by xylose as the second main monosaccharide. An increase in the concentration of commercial cellulase from 500 to 2000 µL could significantly enhance the releasing of monosaccharide from the rice straw waste from 0.2 to 0.8 g/L. However, for the alkali pre-treatment, followed by commercial cellulase, xylose was the predominant monosaccharide. It made up 48–85% of the total monosaccharide compared to glucose that made up 5–49% of total monosaccharide. Also, lower amount of monosaccharide yields were observed in all cases of alkali pre-treatment. Moreover, when the enzyme hydrolysis was performed using the commercial enzyme without any pre-treatment methods, high amount of monosaccharide production was produced at the same concentration range as that of the dilute acid pre-treatment followed by enzymatic hydrolysis method. Glucose was the predominant monosaccharide in this case. Therefore, the best practice approach can be proposed to be the boiled hot water pre-treatment followed by enzymatic hydrolysis. The boiled hot water has potential to penetrate into the structure of rice straw waste and remove mainly hemicellulose and some part of lignin while avoiding formation of inhibitors that occur at high temperatures^30^. In the case of the untreated case followed by enzyme hydrolysis will be the second choice for the field practice. It is interesting that the raw rice straw treated with only commercial cellulase (without pretreatment) could give higher total sugar yields than those obtained from acid and alkali pre-treatments for all dosages of cellulase.

For the reason why the alkali pre-treatment resulted in lower total monosaccharide yields, this might be due to a possible increase in lignin content in the digestate in the alkaline condition as well as the remaining NaOH in the sample that could inhibit the enzyme activity. Indeed, alkaline pre-treatment consists of the solvation of lignocellulose particles and hydrolytic decomposition of lignocellulose. The biomass undergoes swelling, which aids the processes of hydrolysis and fermentation^5^. Pre-treatment with the dilute acid and commercial cellulase gave higher monosaccharides yields than those of alkali pre-treatment for all concentrations of cellulase. More different types of monosaccharides such as glucose, mannose, xylose, and arabinose were found in the case of acid pre-treatment compared to the case of alkaline pre-treatment. Arabinose was not found in the case of alkali pre-treatment. The dilute acid method was reported to be able to hydrolyze the hemicellulose into sugars, however, it cannot dissolve the lignin structure of agricultural residue. Ishizawa et al.^31^ suggested that the dilute acid method could create larger pore volume of the corn stover. Lignocellulosic material is cracked in dilute acid hydrolysis, followed by removal of hemicellulose which resulted in the increased of porosity and enzymatic digestibility of biomass^10^. Therefore, in this study the dilute acid was expected to penetrate to the structure of the rice straw waste and hydrolyze the hemicellulosic part in the lignocellulose structure. This might create the pore structure and increase the release of monosaccharide into the digestate. With the commercial cellulase for raw rice straw with the dilute acid pre-treatment and enzyme hydrolysis, glucose and xylose accounted for 5.27–49.74% and 47.69–84.62% of total monosaccharides, respectively. Similar results were observed for boiled hot water pre-treatment with commercial cellulase at 500 and 1000 µL, glucose and xylose made up to 78.24–83.67% and 9.84–12.14% of total monosaccharides, respectively.

In contrast to the commercial enzyme grade, the pre-treatment with acid and alkali solutions followed by the laboratory grade cellulase gave almost no monosaccharides yields. Small concentrations of total monosaccharides were detected with the unpretreated rice straw with the laboratory grade cellulase enzyme of 7.5 units. However, for boiled hot water pre-treatment, the monosaccharides were also detected even at 2.5 units concentration of laboratory grade cellulase enzyme. This suggested that acid and alkali pre-treatments need higher amount of laboratory grade cellulase for enzymatic hydrolysis. This was a significant drawback of laboratory grade cellulase considering its much higher cost compared to commercial cellulase enzyme.

**Figure 9:**
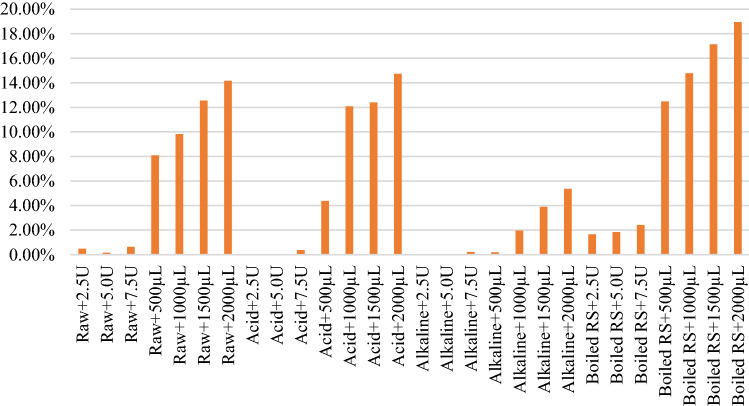
Percentages of Glucose yields.

Figure 9 shows the glucose yields of the untreated and pretreated rice straw followed by enzymatic hydrolysis. The boiled rice straw with commercial cellulase enzyme provided higher glucose yield compared to other experiments (12.48–18.94%) and it can be further increased with an increase in enzyme dosage. It could be inferred that the boiling made rice straw to be more available for enzymatic hydrolysis compared to the chemical pre-treatments at room temperature. According to research reviews, although alkaline pre-treatment has the benefit of increasing digestibility of lignocellulosic biomass, the pre-treatment with either dilute acid or alkaline has limitations on the use of chemicals to optimize pH for enzymatic hydrolysis. The generation of monosaccharides in the liquid digestate was resulted from degradation of the rice straw waste.

Enzymes are used to optimize the production of alternative energy from various substrates. The lignocellulosic rice straw is difficult to be digested by microorganisms.

Therefore, enzymes are introduced to facilitate the degradation of the rice straw waste. Some research works studied the use of cellulase enzyme with the purpose of finding a way to take the benefit from lignocellulosic biomass. Their focus was on the production of sugars such as glucose because it is an important monosaccharide in the production of alternative energy either ethanol or biogas. Examples of studies using cellulase enzymes are shown in Table 3.

**Table 3:** Comparison of pre-treatment methods, enzyme uses, and monosaccharide production.

Enzyme types	Pretreatment Methods	Obtained monosaccharide	References
Spezyme CP (Genencor International, USA) Celluclast 1.5L and Novozyme 188 (Novo, Denmark) Cellulase 100L (Iogen Corporation, Canada) *T. reesei* A1 and *Penicillium* sp. B1 (Fermtech, Russia)	Acidified steam explosion, 160 °C, 10 min H_2_SO_4,_ 160 °C, 10 min AFEX, 1.5 kg, ammonia/kg DM, reactor temp. 74 °C, sample temp. 70 °C, 350 psi, 20 min Enzymatic hydrolysis, 50ºC, 15 min – 72 h	AFEX-pretreated rice straw gave higher carbohydrates conversion Xylose was a significant part (69–70%) followed by arabinose and glucose (20–24%) for AFEX	Vlasenko et al.^32^
Cellucalast 1.5L Novozym 188	Acid pre-treatment, 160-180ºC followed by enzymatic hydrolysis, 50ºC, 72 h	Glucose and xylose release about 83% of the sugar content in the raw material	Hsu et al.^14^
Cellulase enzyme from Genencor International, UAS	Dilute H_2_SO_4_, 20 min followed by enzymatic hydrolysis, 50ºC, 60 h	Total yield of glucose was about 76% of glucose in the raw material	Wei et al.^33^
Cellic Ctec Accelerase 1000TM Accelerase 1500 TM	H_2_SO_4_, 121ºC, 15 min followed by enzymatic hydrolysis, 50ºC, 72 h	The highest sugar and ethanol production was found from the using of Cellic Ctec cellulase	Srinorakutara et al.^34^
Cellulase and laccase enzyme produced from microbial culture	Nitric acid pre-treatment, 160-180ºC followed by microbial degradation at 30ºC, 72 h	An increase in glucose amount in the supernatant was 29.13% as compared to the control samples	Chownk et al.^35^
Cocktails of immobilized enzymes (Celluclast 1.5L, BGL, and laccase)	Dilute sulfuric acid pre-treatment, 121 ºC followed by immobilized enzymatic hydrolysis, 35-55ºC, 48 h	Sugar yields were in the range of 53–67% with acid concentration from 0.1% to 1.0% H2SO4	Kumar et al.^36^
Cellulase enzyme from *Aspergillus Niger* Atres Zymix Plus (Atres group, Germany)	Different integrated enzyme hydrolysis approaches: Dilute H_2_SO_4_, room temp, 4 h Dilute NaOH, room temp, 4 h Boiled water, 4 h Combined with Enzymatic hydrolysis, 50-55ºC, 48 h	Boiled rice straw followed by enzymatic hydrolysis gave the highest monosaccharide yield in the digestate Glucose was the significant part (78–86%) of the monosaccharides	This study

### Insights into monosaccharide production pathway from rice straw waste degradation

**Figure 10:**
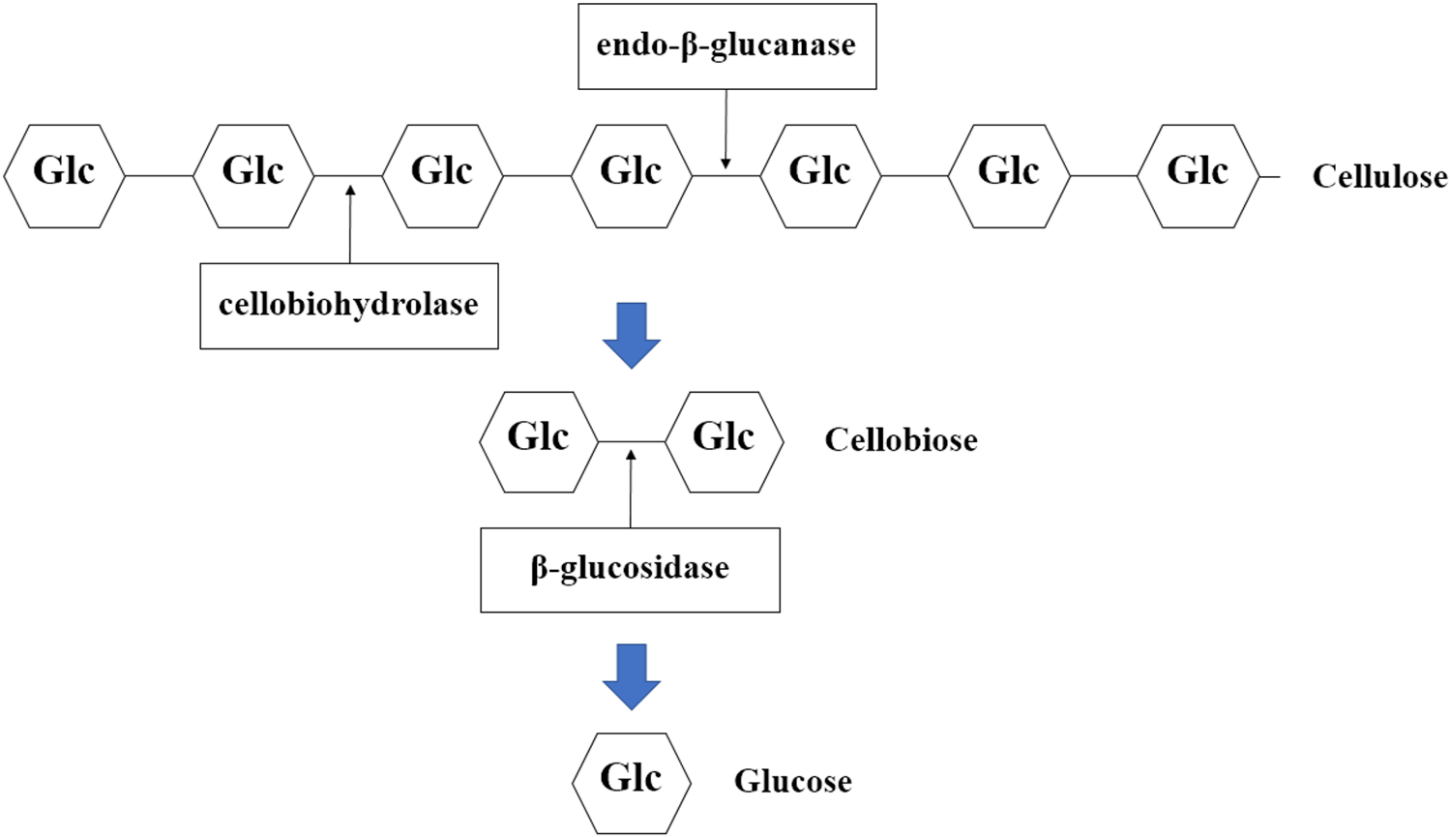
Proposed simplified-diagram of monosaccharide production from the cellulose degradation of the rice straw waste (adapted from Kumar et al.^36^).

The obtained monosaccharides were produced from the degradation of cellulose and hemicellulose in the rice straw waste. Cellulose degradation provided only glucose because cellulose was composed of only D-glucose units linked by β-(1,4)-glycosidic bonds. The smallest repetitive unit of cellulose was cellobiose. Cellobiose, was degraded from cellulose by endo-β-glucan and cellobiohydrolase enzyme^36^. Since the cellulase enzyme used in this research consists of a group of enzymes, which include endo-β-glucan and cellobiohydrolase enzymes; therefore, the cellulase enzyme can convert cellulose to glucose as the main final product as shown in Fig. 10.

**Figure 11:**
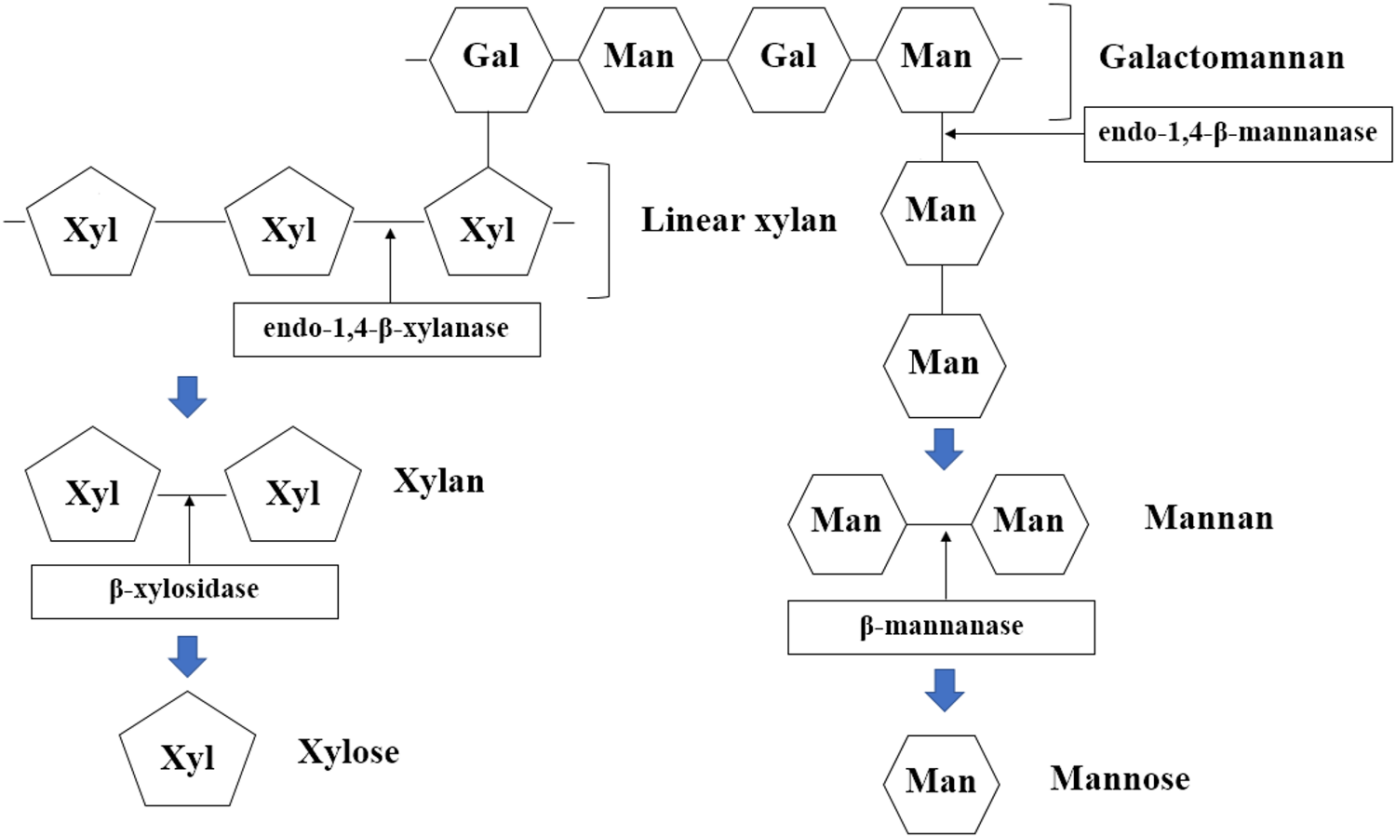
Proposed simplified-diagram of monosaccharide production from hemicellulose degradation of the rice straw waste.

Hemicelluloses are a group of branched polymers. There are built up by both pentose sugars (D-xylose, L-arabinose) and hexose sugars (D-mannose, D-glucose, D-galactose) and sugar acids. They are easily hydrolysed due to their branched structures. Xylan is the second most abundant hemicellulosic polysaccharides in nature. It is comprised of xylose units linked by β-(1,4)-glycosidic bonds. There are many different enzymes involved in monosaccharide production from hemicellulose degradation of the rice straw waste as shown in Fig. 11. Since the commercial enzyme is a mixture of enzyme groups such as cellulase, xylanase, endo-1,4, it can further degrade the hemicellulose into Xylose and Mannose as the final main products.

**Figure 12:**
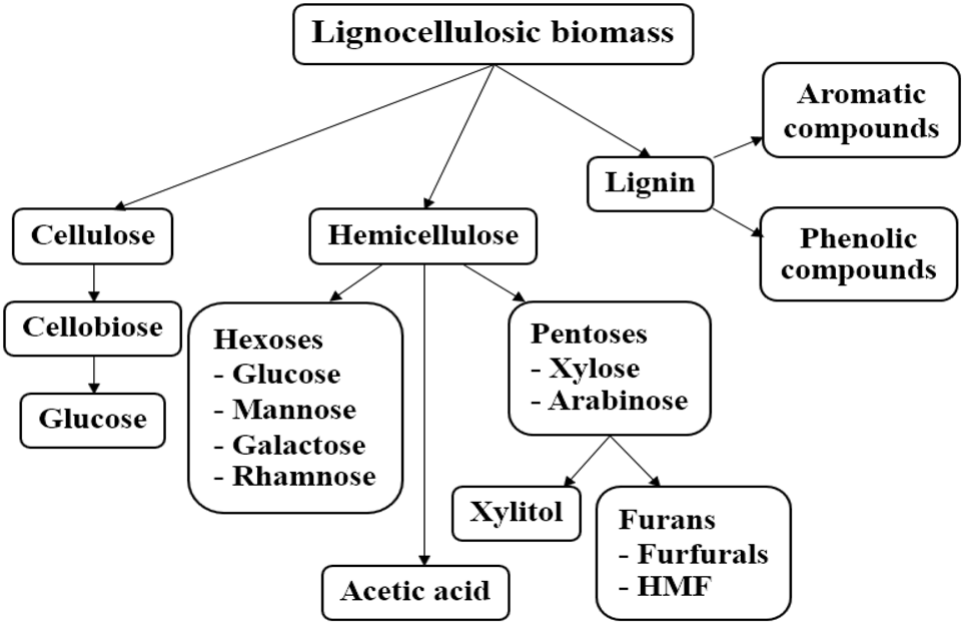
Proposed pathway of monosaccharide production from lignocellulosic degradation of the rice straw waste.

The monosaccharides produced from cellulose and hemicellulose degradation, as fermentable sugars, was consumed by microorganisms and transformed into biofuels production such as ethanol and biogas. The obtained result from this study shows that lignocellulosic biomass was degraded through various pre-treatments and digested by cellulase enzyme in the integrated enzymatic hydrolysis methods. Cellulose, hemicellulose, and lignin are the main structures of lignocellulosic cellulose. Figure 12 illustrates the proposed pathway of monosaccharide production from lignocellulosic degradation of the rice straw waste. The possible products from the degradation are also described. Hexose is a simple sugar with six carbon atoms. The chemical formula of all hexose is C_6_H_12_O_6_. Morrison and Boyd^28^ stated that two important hexoses in nature are glucose and fructose. Like pentoses, hexoses exist in both open-chain form and cyclic form which can be easily converted into each other in aqueous solution. Cellulose consists of units of hexose (glucose), while both pentose and hexose sugars are found in hemicellulose structure.

This work suggested that most of the by-products were volatile organic substances which can be identified by GC–MS/MS such as furan derivatives, phenolic compounds, aromatic hydrocarbons, or acetic acids. It can be inferred that rice straw is a lignocellulosic biomass with high potential for production in many industries. Acetic acid was released from every pre-treatment technique. Phenolic compounds such as tannic acid and vanillin which can be enzymatic hydrolyzed and microorganism activity inhibitors were not found in this research even with the boiled hot water pre-treatment method.

## Conclusions

Rice straw is one of the abundant lignocellulosic biomasses which can be considered as a renewable energy source. The pathway of monosaccharide production from lignocellulosic degradation of the Hom–Mali Rice straw waste was proposed in this study from the integrated enzymatic hydrolysis methods. Total monosaccharide yields were the major consideration for selecting suitable pre-treatments for enzymatic hydrolysis. The integrated enzymatic hydrolysis with boiled hot water pre-treatment could achieve the highest monosaccharide production from the rice straw waste. Hydrolysis of the rice straw waste with the commercial cellulase gave slightly lower monosaccharide yields compared to the boiled hot water pre-treatment followed by enzymatic hydrolysis. Hydrothermal pre-treatment or hot water pre-treatment before adding enzyme is an environmental-friendly option because it does not need chemicals to neutralize. For the future implementation, the commercialized digestion plants should utilize the waste heat from engine generator set, that is commonly available. Therefore, the waste heat can be utilized for hot water pre-treatment. Enzymatic hydrolysis of raw rice straw using cellulase without any pre-treatment is also effective if it is needed to avoid the added cost of waste heat utilization for hot water pre-treatment.

## Supplementary Information

Supplementary Information.

## Data Availability

All data is included in this article. There is no supplemental data available in this article.
