# Supplementary material for: Insight into pathway of monosaccharide production from integrated enzymatic hydrolysis of rice straw waste as feed stock for anaerobic digestion
Source: Sci Rep. 2023 Jan 4;13:148. doi: 10.1038/s41598-023-27398-6 (PMC9813138; doi:10.1038/s41598-023-27398-6)
Supplement: Supplementary file 1 — Supplementary Information. [file 41598_2023_27398_MOESM1_ESM.docx]

HIGHLIGHTS

- Lack of data on monosaccharide production from rice straw waste degradation (Thai Hom-Mali Rice).
- Platform of monosaccharide production was investigated to know the enzyme efficiency.
- This research proposed the possible pathway of monosaccharide production from rice straw waste.
- Three integrated enzymatic hydrolysis methods were performed to know the hydrolysis products.
- Boiled rice straw with commercial enzyme provided the highest monosaccharide yield.
- Aromatic and aliphatic hydrocarbon substances were also detected in the FEEM analysis.
